# Trisilver(I) citrate

**DOI:** 10.1107/S160053681100239X

**Published:** 2011-01-22

**Authors:** Andreas Fischer

**Affiliations:** a Inorganic Chemistry, School of Chemical Science and Engineering, Royal Institute of Technology (KTH), 100 44 Stockholm, Sweden

## Abstract

Trisilver(I) citrate, 3Ag^+^·C_6_H_5_O_7_^3−^, was obtained by evaporation of a saturated aqueous solution of the raw material that had been obtained from sodium dihydrogen citrate and silver nitrate. It features one formula unit in the asymmetric unit. There is an intra­molecular O—H⋯O hydrogen bond between the OH group and one of the terminal carboxyl­ate groups. Different citrate groups are linked *via* the three Ag^+^ ions, yielding a three-dimensional network with rather irregular [AgO_4_] polyhedra.

## Related literature

For the preparation and structure of ammonium disilver(I) citrate monohydrate, see: Sagatys *et al.* (1993 ▶) and for tetra­ammonium copper(II) bis­(citrate), see: Bott *et al.* (1991 ▶). For ^109^Ag solid-state NMR studies on different silver salts, including commercial silver citrate, see: Penner & Li (2004 ▶).
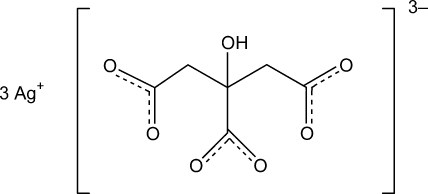


## Experimental

### 

#### Crystal data

3Ag^+^·C_6_H_5_O_7_^3−^
*M* *_r_* = 512.71
Orthorhombic, 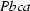

*a* = 6.6181 (7) Å
*b* = 11.8477 (11) Å
*c* = 22.386 (2) Å
*V* = 1755.3 (3) Å^3^
*Z* = 8
Mo *K*α radiation
μ = 6.65 mm^−1^
*T* = 299 K
0.12 × 0.05 × 0.02 mm

#### Data collection

Bruker–Nonius KappaCCD diffractometer
Absorption correction: multi-scan (*SADABS*; Sheldrick, 2003 ▶) *T*_min_ = 0.631, *T*_max_ = 0.876
15238 measured reflections
2008 independent reflections
1493 reflections with *I* > 2σ(*I*)
*R*_int_ = 0.055

#### Refinement

*R*[*F* ^2^ > 2σ(*F* ^2^)] = 0.032
*wR*(*F* ^2^) = 0.051
*S* = 1.10
2008 reflections
148 parameters
1 restraint
H atoms treated by a mixture of independent and constrained refinement
Δρ_max_ = 1.21 e Å^−3^
Δρ_min_ = −1.21 e Å^−3^

### 

Data collection: *COLLECT* (Nonius, 1998 ▶); cell refinement: *DIRAX* (Duisenberg, 1992 ▶); data reduction: *EVALCCD* (Duisenberg *et al.*, 2003 ▶); program(s) used to solve structure: *SHELXS97* (Sheldrick, 2008 ▶); program(s) used to refine structure: *SHELXL97* (Sheldrick, 2008 ▶); molecular graphics: *DIAMOND* (Brandenburg, 2007) ▶; software used to prepare material for publication: *publCIF* (Westrip, 2010 ▶).

## Supplementary Material

Crystal structure: contains datablocks global, I. DOI: 10.1107/S160053681100239X/kp2301sup1.cif

Structure factors: contains datablocks I. DOI: 10.1107/S160053681100239X/kp2301Isup2.hkl

Additional supplementary materials:  crystallographic information; 3D view; checkCIF report

## Figures and Tables

**Table 1:** Selected bond lengths (Å)

Ag1—O6^i^	2.275 (3)
Ag1—O3	2.416 (3)
Ag1—O6^ii^	2.539 (3)
Ag1—O7^ii^	2.555 (3)
Ag2—O2^iii^	2.300 (3)
Ag2—O3	2.477 (4)
Ag2—O7^ii^	2.550 (3)
Ag2—O2^ii^	2.566 (3)
Ag3—O4	2.197 (3)
Ag3—O1^iii^	2.340 (3)
Ag3—O5^iv^	2.404 (3)
Ag3—O4^iv^	2.519 (4)

Symmetry codes: (i) 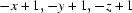; (ii) 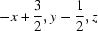; (iii) 
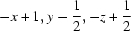
; (iv) 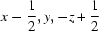.

**Table 2:** Hydrogen-bond geometry (Å, °)

*D*—H⋯*A*	*D*—H	H⋯*A*	*D*⋯*A*	*D*—H⋯*A*
O5—H5*O*⋯O7	0.81 (2)	1.90 (3)	2.636 (5)	152 (5)

**Fig. 1.**
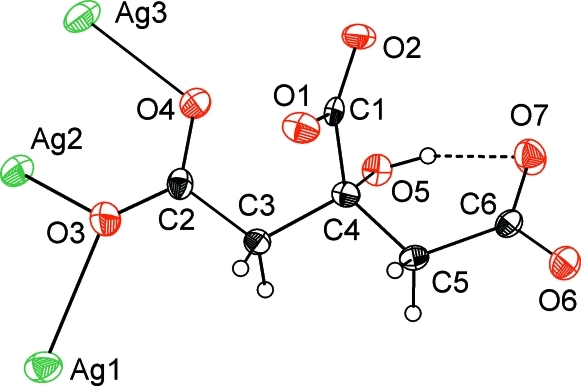
The molecular structure of the title compound. Thermal ellipsoids at the 50% probability level. H bond as dashed line.

## supplementary crystallographic information

### Comment

The structures of many citrates of common metal ions are surprisingly sparsely investigated. Of the citrates of coinage metal cations, only ammonium disilver citrate monohydrate (Sagatys *et al.*, 1993) and tetraammonium copper(II) bis(citrate) (Bott *et al.* 1991) have been reported. Here, we report the crystal structure of trisilver citrate, which was obtained from mixing solutions of sodium dihydrogen citrate and silver nitrate.

The basic structural of the 3D-polymeric structure shows an intramolecular hydrogen bond O–H···O bond between O5 and O7 (Fig. 1). As expected from the charges, all three carboxy groups are deprotonated. The coordination polyhedra about the Ag^+^ cations are quite irregular, the Ag–O distances are in the range 2.275 (3) to 2.566 (3) Å (Table 1). The  Ag2–Ag3 contact of 2.8801 (6) Å is the shortest one  in the structure. It can be noted that significantly shorter distances Ag^+^–Ag^+^ are observed in many silver coordination compounds.

### Experimental

An aqueous solution (0.5 mol/L) of sodium dihydrogen citrate was prepared by dissolving the respecitve amounts of trisodium citrate (Merck, p.a.) and citric acid in demineralised water. 1 mL of this solution was added to 1 mL of a solution of silver nitrate (0.5 mol/L), yielding a white precipitate. The latter was washed with demineralised water. The precipitate was then heated to 323 K with 1 mL of demineralised water. Upon cooling to room remperature, the saturated solution was filtered off and put aside for evaporation. Within a couple of days, small, colourless, rod-like crystals formed, that were suitable for structure determination. It can be noted that crystals of the title compound turned brown during the structure determination; however, no significant decrease in diffraction intensity could be observed. The initial precipitate formed from sodium dihydrogen citrate and silver nitrate was investigated by powder diffraction and it could be confirmed that it consisted of pure trisilver citrate. In addition, this powder pattern is identical with that of commercial "silver citrate hydrate".

### Refinement

Methylene-H atoms were placed at calculated positions (C–H=0.97 Å, *U_iso_*=1.2 *U_eq_* of the respective C atom). The hydroxy-H atom was located from the Fourier map and was refined with a restraint (O–H=0.82 (2) Å) and *U_iso_*=1.5 *U_eq_*(O)). The largest Fourier peak/hole (1.21 and -1.21 e/Å^3^, respectively), are found 0.82 and 0.77Å from Ag2.

### Crystal data

**Table:**

3Ag^+^·C_6_H_5_O_7_^3^^−^	*F*(000) = 1904
*M**_r_* = 512.71	*D*_x_ = 3.880 Mg m^−^^3^
Orthorhombic, *P**b**c**a*	Mo *K*α radiation, λ = 0.71073 Å
Hall symbol: -P 2ac 2ab	Cell parameters from 53 reflections
*a* = 6.6181 (7) Å	θ = 4.0–20.0°
*b* = 11.8477 (11) Å	µ = 6.65 mm^−^^1^
*c* = 22.386 (2) Å	*T* = 299 K
*V* = 1755.3 (3) Å^3^	Rod, colourless
*Z* = 8	0.12 × 0.05 × 0.02 mm

### Data collection

**Table:**

Bruker–Nonius KappaCCD diffractometer	1493 reflections with *I* > 2σ(*I*)
Radiation source: fine-focus sealed tube	*R*_int_ = 0.055
φ & ω scans	θ_max_ = 27.5°, θ_min_ = 4.6°
Absorption correction: multi-scan (*SADABS*; Sheldrick, 2003)	*h* = −8→8
*T*_min_ = 0.631, *T*_max_ = 0.876	*k* = −14→15
15238 measured reflections	*l* = −29→29
2008 independent reflections	

### Refinement

**Table:**

Refinement on *F*^2^	Primary atom site location: structure-invariant direct methods
Least-squares matrix: full	Secondary atom site location: difference Fourier map
*R*[*F*^2^ > 2σ(*F*^2^)] = 0.032	H atoms treated by a mixture of independent and constrained refinement
*wR*(*F*^2^) = 0.051	*w* = 1/[σ^2^(*F*_o_^2^) + (0.0117*P*)^2^ + 5.8189*P*] where *P* = (*F*_o_^2^ + 2*F*_c_^2^)/3
*S* = 1.10	(Δ/σ)_max_ = 0.001
2008 reflections	Δρ_max_ = 1.21 e Å^−^^3^
148 parameters	Δρ_min_ = −1.21 e Å^−^^3^
1 restraint	

### Special details

**Table:**

Geometry. All e.s.d.'s (except the e.s.d. in the dihedral angle between two l.s. planes) are estimated using the full covariance matrix. The cell e.s.d.'s are taken into account individually in the estimation of e.s.d.'s in distances, angles and torsion angles; correlations between e.s.d.'s in cell parameters are only used when they are defined by crystal symmetry. An approximate (isotropic) treatment of cell e.s.d.'s is used for estimating e.s.d.'s involving l.s. planes.
Refinement. Refinement of *F*^2^ against ALL reflections. The weighted *R*-factor *wR* and goodness of fit *S* are based on *F*^2^, conventional *R*-factors *R* are based on *F*, with *F* set to zero for negative *F*^2^. The threshold expression of *F*^2^ > σ(*F*^2^) is used only for calculating *R*-factors(gt) *etc*. and is not relevant to the choice of reflections for refinement. *R*-factors based on *F*^2^ are statistically about twice as large as those based on *F*, and *R*- factors based on ALL data will be even larger.

### Fractional atomic coordinates and isotropic or equivalent isotropic displacement parameters (Å^2^)

**Table:**

	*x*	*y*	*z*	*U*_iso_*/*U*_eq_	
Ag1	0.10659 (6)	0.25916 (4)	0.431564 (16)	0.02802 (12)	
Ag2	0.27992 (6)	0.15476 (4)	0.296424 (16)	0.03187 (12)	
Ag3	0.42015 (6)	0.32800 (4)	0.216249 (16)	0.02955 (12)	
C1	0.7295 (7)	0.5962 (4)	0.33652 (19)	0.0165 (10)	
C2	0.4709 (7)	0.3885 (4)	0.3454 (2)	0.0181 (11)	
C3	0.5610 (7)	0.4467 (4)	0.39995 (19)	0.0168 (10)	
C4	0.7567 (7)	0.5110 (4)	0.38916 (18)	0.0155 (10)	
C5	0.8155 (7)	0.5769 (4)	0.44573 (19)	0.0160 (10)	
C6	1.0229 (7)	0.6305 (4)	0.4444 (2)	0.0176 (11)	
O1	0.5862 (5)	0.6651 (3)	0.34287 (14)	0.0244 (8)	
O2	0.8489 (5)	0.5926 (3)	0.29317 (14)	0.0240 (8)	
O3	0.3092 (5)	0.3355 (3)	0.35141 (15)	0.0317 (9)	
O4	0.5631 (5)	0.3974 (3)	0.29684 (14)	0.0307 (9)	
O5	0.9113 (5)	0.4296 (3)	0.37668 (14)	0.0186 (7)	
O6	1.0678 (5)	0.7019 (3)	0.48333 (15)	0.0296 (9)	
O7	1.1480 (5)	0.6006 (3)	0.40461 (15)	0.0272 (8)	
H3A	0.4617	0.4990	0.4157	0.020*	
H3B	0.5856	0.3900	0.4304	0.020*	
H5A	0.7161	0.6359	0.4522	0.019*	
H5B	0.8089	0.5260	0.4796	0.019*	
H5O	1.012 (5)	0.468 (4)	0.379 (2)	0.028*	

### Atomic displacement parameters (Å^2^)

**Table:**

	*U*^11^	*U*^22^	*U*^33^	*U*^12^	*U*^13^	*U*^23^
Ag1	0.0264 (2)	0.0362 (3)	0.02149 (19)	0.00746 (19)	0.00281 (17)	−0.00024 (17)
Ag2	0.0284 (2)	0.0484 (3)	0.01882 (19)	−0.0047 (2)	−0.00075 (17)	−0.00654 (19)
Ag3	0.0383 (3)	0.0327 (3)	0.01763 (18)	−0.0018 (2)	−0.00653 (17)	−0.00150 (17)
C1	0.014 (2)	0.020 (3)	0.016 (2)	−0.005 (2)	−0.004 (2)	−0.003 (2)
C2	0.018 (3)	0.014 (3)	0.022 (2)	−0.002 (2)	−0.004 (2)	0.002 (2)
C3	0.014 (2)	0.021 (3)	0.015 (2)	0.001 (2)	0.0015 (19)	−0.0013 (19)
C4	0.015 (2)	0.016 (3)	0.016 (2)	0.003 (2)	0.001 (2)	0.0021 (19)
C5	0.019 (3)	0.015 (3)	0.014 (2)	0.002 (2)	0.0004 (19)	0.0012 (19)
C6	0.017 (3)	0.020 (3)	0.016 (2)	0.002 (2)	−0.004 (2)	0.003 (2)
O1	0.0250 (19)	0.023 (2)	0.0250 (17)	0.0080 (17)	0.0029 (15)	0.0076 (15)
O2	0.0222 (18)	0.034 (2)	0.0162 (16)	0.0004 (16)	0.0039 (15)	0.0037 (15)
O3	0.029 (2)	0.040 (2)	0.0260 (18)	−0.013 (2)	0.0013 (16)	−0.0050 (17)
O4	0.029 (2)	0.044 (2)	0.0188 (17)	−0.0117 (18)	0.0016 (17)	−0.0080 (16)
O5	0.0197 (19)	0.0140 (19)	0.0220 (16)	0.0030 (16)	0.0001 (15)	−0.0033 (14)
O6	0.031 (2)	0.034 (2)	0.0232 (17)	−0.0112 (18)	0.0018 (16)	−0.0115 (16)
O7	0.0223 (19)	0.030 (2)	0.0296 (19)	−0.0022 (17)	0.0038 (16)	−0.0077 (16)

### Geometric parameters (Å, °)

**Table:**

Ag1—O6^i^	2.275 (3)	C3—C4	1.522 (6)
Ag1—O3	2.416 (3)	C4—O5	1.433 (6)
Ag1—O6^ii^	2.539 (3)	C4—C5	1.538 (6)
Ag1—O7^ii^	2.555 (3)	C5—C6	1.513 (7)
Ag2—O2^iii^	2.300 (3)	C6—O6	1.251 (6)
Ag2—O3	2.477 (4)	C6—O7	1.266 (6)
Ag2—O7^ii^	2.550 (3)	O1—Ag3^vi^	2.340 (3)
Ag2—O2^ii^	2.566 (3)	O2—Ag2^vi^	2.300 (3)
Ag2—Ag3	2.8801 (6)	O2—Ag2^vii^	2.566 (3)
Ag2—Ag3^iv^	3.1563 (7)	O4—Ag3^v^	2.519 (4)
Ag3—O4	2.197 (3)	O5—Ag3^v^	2.404 (3)
Ag3—O1^iii^	2.340 (3)	O6—Ag1^i^	2.275 (3)
Ag3—O5^iv^	2.404 (3)	O6—Ag1^vii^	2.539 (3)
Ag3—O4^iv^	2.519 (4)	O7—Ag2^vii^	2.550 (3)
Ag3—Ag2^v^	3.1563 (7)	O7—Ag1^vii^	2.555 (3)
C1—O2	1.252 (5)	C3—H3A	0.9700
C1—O1	1.260 (6)	C3—H3B	0.9700
C1—C4	1.562 (6)	C5—H5A	0.9700
C2—O3	1.248 (6)	C5—H5B	0.9700
C2—O4	1.252 (6)	O5—H5O	0.81 (2)
C2—C3	1.523 (6)		
			
O6^i^—Ag1—O3	145.89 (13)	O4—C2—C3	117.8 (4)
O6^i^—Ag1—O6^ii^	95.88 (6)	C4—C3—C2	115.6 (4)
O3—Ag1—O6^ii^	88.17 (12)	O5—C4—C3	107.6 (4)
O6^i^—Ag1—O7^ii^	132.00 (12)	O5—C4—C5	108.8 (4)
O3—Ag1—O7^ii^	75.34 (12)	C3—C4—C5	109.8 (3)
O6^ii^—Ag1—O7^ii^	51.10 (11)	O5—C4—C1	111.7 (3)
O2^iii^—Ag2—O3	137.63 (12)	C3—C4—C1	110.2 (4)
O2^iii^—Ag2—O7^ii^	144.77 (12)	C5—C4—C1	108.8 (4)
O3—Ag2—O7^ii^	74.39 (11)	C6—C5—C4	115.2 (4)
O2^iii^—Ag2—O2^ii^	103.78 (10)	O6—C6—O7	121.6 (4)
O3—Ag2—O2^ii^	100.80 (12)	O6—C6—C5	119.1 (4)
O7^ii^—Ag2—O2^ii^	77.04 (10)	O7—C6—C5	119.4 (4)
O2^iii^—Ag2—Ag3	78.71 (9)	C1—O1—Ag3^vi^	119.0 (3)
O3—Ag2—Ag3	70.64 (8)	C1—O2—Ag2^vi^	115.5 (3)
O7^ii^—Ag2—Ag3	135.29 (8)	C1—O2—Ag2^vii^	124.9 (3)
O2^ii^—Ag2—Ag3	83.01 (8)	Ag2^vi^—O2—Ag2^vii^	106.72 (12)
O2^iii^—Ag2—Ag3^iv^	81.39 (9)	C2—O3—Ag1	138.1 (3)
O3—Ag2—Ag3^iv^	62.71 (9)	C2—O3—Ag2	116.7 (3)
O7^ii^—Ag2—Ag3^iv^	112.94 (8)	Ag1—O3—Ag2	90.13 (12)
O2^ii^—Ag2—Ag3^iv^	155.01 (8)	C2—O4—Ag3	118.2 (3)
Ag3—Ag2—Ag3^iv^	73.958 (16)	C2—O4—Ag3^v^	122.1 (3)
O4—Ag3—O1^iii^	141.33 (13)	Ag3—O4—Ag3^v^	100.73 (13)
O4—Ag3—O5^iv^	122.24 (13)	C4—O5—Ag3^v^	121.5 (2)
O1^iii^—Ag3—O5^iv^	85.60 (11)	C6—O6—Ag1^i^	126.9 (3)
O4—Ag3—O4^iv^	112.16 (13)	C6—O6—Ag1^vii^	93.7 (3)
O1^iii^—Ag3—O4^iv^	100.76 (12)	Ag1^i^—O6—Ag1^vii^	139.37 (15)
O5^iv^—Ag3—O4^iv^	73.33 (11)	C6—O7—Ag2^vii^	136.0 (3)
O4—Ag3—Ag2	83.90 (9)	C6—O7—Ag1^vii^	92.6 (3)
O1^iii^—Ag3—Ag2	76.06 (8)	Ag2^vii^—O7—Ag1^vii^	85.45 (11)
O5^iv^—Ag3—Ag2	152.65 (8)	C4—C3—H3A	108.4
O4^iv^—Ag3—Ag2	90.16 (8)	C2—C3—H3A	108.4
O4—Ag3—Ag2^v^	89.56 (10)	C4—C3—H3B	108.4
O1^iii^—Ag3—Ag2^v^	55.00 (8)	C2—C3—H3B	108.4
O5^iv^—Ag3—Ag2^v^	105.44 (8)	H3A—C3—H3B	107.4
O4^iv^—Ag3—Ag2^v^	155.43 (9)	C6—C5—H5A	108.5
Ag2—Ag3—Ag2^v^	80.542 (17)	C4—C5—H5A	108.5
O2—C1—O1	125.8 (4)	C6—C5—H5B	108.5
O2—C1—C4	119.4 (4)	C4—C5—H5B	108.5
O1—C1—C4	114.9 (4)	H5A—C5—H5B	107.5
O3—C2—O4	123.6 (4)	C4—O5—H5O	101 (4)
O3—C2—C3	118.6 (4)	Ag3^v^—O5—H5O	109 (4)

Symmetry codes: (i) −*x*+1, −*y*+1, −*z*+1; (ii) −*x*+3/2, *y*−1/2, *z*; (iii) −*x*+1, *y*−1/2, −*z*+1/2; (iv) *x*−1/2, *y*, −*z*+1/2; (v) *x*+1/2, *y*, −*z*+1/2; (vi) −*x*+1, *y*+1/2, −*z*+1/2; (vii) −*x*+3/2, *y*+1/2, *z*.

### Hydrogen-bond geometry (Å, °)

**Table:**

*D*—H···*A*	*D*—H	H···*A*	*D*···*A*	*D*—H···*A*
O5—H5O···O7	0.81 (2)	1.90 (3)	2.636 (5)	152 (5)
